# Pixel modelling – a new age in SFX data analysis

**DOI:** 10.1107/S2052252520014281

**Published:** 2020-10-30

**Authors:** Karol Nass

**Affiliations:** a SwissFEL, Paul Scherrer Institut, Forschungsstrasse 111, Villigen PSI, 5232, Switzerland

**Keywords:** serial crystallography, XFELs, data analysis, maximum likelihood

## Abstract

A new program, *diffBragg*, employs per-pixel maximum likelihood optimization of X-ray pulse and crystal parameters to improve the accuracy of structure factor amplitudes attainable in SFX experiments.

Over a decade ago, the first proof-of-principle serial femtosecond crystallography (SFX) experiment started the era of nano- and micro-crystallography at X-ray free-electron lasers (XFELs) (Chapman *et al.*, 2011 ▸). The high peak brilliance and femtosecond duration of the XFEL pulses enabled the long-awaited radiation-damage-free data collection at room temperature from fully hydrated samples (Nass, 2019 ▸). This opened up unmatched opportunities for structural studies of radiation sensitive metalloproteins (Kern *et al.*, 2015 ▸; Suga *et al.*, 2020 ▸) and small, weakly diffracting protein crystals, including membrane proteins (Johansson *et al.*, 2017 ▸).

Moreover, the femtosecond XFEL pulse duration allows light to be shed on the structure–function relationship of photo-activated proteins by enabling sub-picosecond time-resolution in optical-pump X-ray-probe SFX studies (Orville, 2020 ▸). In this case, small crystal sizes are a necessity due to the limited penetration depth of the pump laser in protein crystals. By combining radiation-damage-free data collection from tiny crystals at room temperature with the sub-picosecond time-resolution in pump-probe experiments, SFX is a very powerful tool in the hands of structural biologists (Spence, 2017 ▸).

However, despite continuous efforts to improve state-of-the art SFX, it still suffers from major technical challenges that limit the throughput and accessibility of this relatively new technique, especially for non-expert user groups. These challenges occur in all aspects of the experiment, starting with radically different sample requirements (Beale *et al.*, 2019 ▸), non-standardized methods and complex sample delivery equipment (Grünbein & Nass Kovacs, 2019 ▸), and finishing with demanding and heavily supervised data collection and analysis that requires significant computational resources.

In particular, analysis of the SFX data is complex. A fundamental limitation is the femtosecond pulse duration precluding rotation of the crystal during exposure, which results in measuring diffraction patterns from still crystals in random orientations that contain only partially integrated reflections. Moreover, it suffers from many sources of errors due to the stochastic nature of XFEL pulses and morphological differences between measured crystals. The XFEL pulse instability affects shot-to-shot intensity, width and centre of the wavelength spread and the exact shape of energy spectrum. On the sample side, the distribution of crystal sizes and unit-cell dimensions and the degree of non-isomorphism and mosaicity contribute to the uncertainty in SFX data analysis. All this makes assembly of a complete set of full and accurate structure factor amplitudes for molecular replacement strategies challenging, and even more so for *de novo* structure determination (Nass *et al.*, 2016 ▸).

To date, the most popular SFX data processing programs use the so-called ‘Monte Carlo’ method, which is based on averaging partial intensity measurements of equivalent reflections from many different crystals. This method can decrease the contribution of random sources of errors described above to the structure factor amplitudes, but it is inefficient in terms of sample and beam time usage, as it requires tens of thousands of indexed diffraction patterns to converge (Kirian *et al.*, 2010 ▸). Extension of this ‘Monte Carlo’ method with post-refinement and partiality corrections recently provided tangible improvements to the determination of accurate anomalous structure factor differences required for *de novo* phasing by needing significantly fewer diffraction images than before (Nass *et al.*, 2020 ▸). However, these methods still rely on integration of the Bragg spot intensities located under globally defined, fixed-width areas predicted by incomplete diffraction models without taking into account the shape and size of the spots (Fig. 1 ▸).

In this issue of **IUCrJ**, Mendez and co-workers (Mendez *et al.*, 2020 ▸) present a new data analysis approach, *diffBragg*, that proposes to increase the accuracy of structure factor amplitudes attainable in SFX experiments. In contrast to the ‘Monte Carlo’ approach defined in Kirian *et al.*, *diffBragg* employs an elaborated physical model and maximum likelihood estimation to describe the intensity of all observed pixels in Bragg spots and in their vicinity across all images. As opposed to other SFX data processing programs, the model presented by the authors takes into account most of the important factors that determine the intricate shapes, sizes and intensity profiles of Bragg spots to improve accuracy of final structure factor amplitudes. The parameters optimized by the pixel modelling include crystal orientation, unit-cell parameters, intensity scale factor, mosaic parameters, incident photon spectra and a starting list of structure factor amplitudes provided by an initial round of conventional data processing.

This work paves the way for next-generation SFX data analysis by enabling to decouple contributions of these various experimental sources or error from the measured Bragg spot intensities, which otherwise obscure structure factor amplitudes determination. The authors achieve an order of magnitude reduction in the number of required diffraction images yielding the same accuracy of the anomalous structure factor amplitudes, as compared to the conventional ‘Monte Carlo’ approach. Although these improvements were demonstrated with simulated diffraction data of ytterbium derivative lysozyme crystals, the method was shown to be robust against many experimental sources of error (*e.g.* background scattering, measurement noise, typical detector panel displacement). This indicates that *diffBragg* could have a remarkable impact on future SFX experiments by addressing one of the main bottlenecks of SFX – the need for high amounts of data, translating into large sample quantities. Further, the high accuracy achieved with pixel-level refinement can provide a clear view on extremely sensitive details such as two differently oxidized metal atoms (Sauter *et al.*, 2020 ▸). The ability to observe such level of detail expands opportunities for new and exciting experiments to consider for the upcoming years of SFX science.

## Figures and Tables

**Figure 1:**
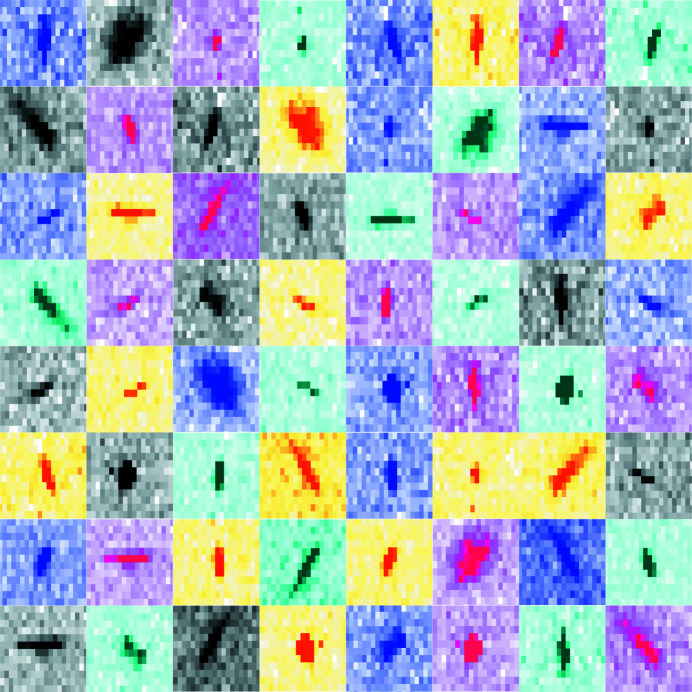
Collage of partial Bragg spots recorded during SFX experiments. For each pixel in the ‘shoe-box’ of a given Bragg spot, *diffBragg* applies precise modelling of the various parameters affecting their shape and intensity, such as lattice orientation, unit-cell dimensions, mosaic structure, incident photon spectra and partiality.
